# Modulation of Oxidative Stress and Nuclear Factor-κB–Associated Inflammatory Signaling by Rabeprazole in Acetic Acid–Induced Colonic Injury

**DOI:** 10.5152/tjg.2026.26033

**Published:** 2026-05-20

**Authors:** Aykut Özbek, Mehmet Yıldız, Engin Altınkaya, Halef Okan Doğan, Mustafa Özkaraca, Hilmi Ataseven

**Affiliations:** 1 Department of Internal Medicine, Sivas Cumhuriyet University Faculty of Medicine, Sivas, Türkiye; 2 Division of Gastroenterology, Department of Internal Medicine, Sivas Cumhuriyet University Faculty of Medicine, Sivas, Türkiye; 3 Department of Biochemistry, Sivas Cumhuriyet University Faculty of Medicine, Sivas, Türkiye; 4 Department of Pathology, Sivas Cumhuriyet University Veterinary Faculty, Sivas, Türkiye

**Keywords:** Acetic acid–induced colonic injury, inflammation, oxidative stress, proton pump inhibitors, rabeprazole

## Abstract

**Background/Aims:**

Proton pump inhibitors have been reported to exert anti-inflammatory and antioxidant effects independent of gastric acid suppression; however, their role in inflammatory bowel disease remains unclear. The current study investigated whether rabeprazole modulates oxidative stress–related and inflammatory pathways in an acetic acid–induced colonic injury (AAIC) and compared these effects with those of prednisolone.

**Materials and Methods:**

Forty male Wistar albino rats were randomly assigned to 5 groups (n = 8) using a computer-generated sequence. To ensure unbiased results, the control and AAIC groups received sham gavage of 1 mL of 0.9% NaCl on the same schedule as the treatment groups. Colonic injury was induced by transrectal administration of 3% acetic acid. Rabeprazole (20 mg/kg/day) and/or prednisolone (2 mg/kg/day) were administered orally for 10 days. Macroscopic and histopathological evaluations were performed. Immunohistochemical expression of 8-hydroxy-2′-deoxyguanosine (8-OHdG), transforming growth factor-β (TGF-β), tumor necrosis factor-α (TNF-α), and nuclear factor-κB (NF-κB) was assessed. Serum malondialdehyde (MDA), glutathione, superoxide dismutase (SOD), and catalase (CAT) levels were measured.

**Results:**

Rabeprazole significantly attenuated histopathological damage and inflammatory cell infiltration compared with the AAIC group. Expression of 8-OHdG, TGF-β, TNF-α, and NF-κB was reduced, accompanied by decreased MDA levels and increased SOD and CAT activities. These effects overlapped with those of prednisolone. Combined administration did not provide additional benefit and was associated with reduced protective responses compared with either agent alone.

**Conclusion:**

Rabeprazole modulates oxidative stress and inflammatory pathways and alleviates tissue injury in experimental colonic injury; however, its effects are not additive with those of prednisolone. Further studies are warranted to clarify the underlying mechanisms.

**Main Points**

- Rabeprazole reduced histopathological damage and inflammatory cell infiltration in an experimental model of colonic injury.
- Rabeprazole attenuated oxidative DNA damage, as reflected by decreased 8-hydroxy-2′-deoxyguanosine expression.
- Suppression of nuclear factor-κB and tumor necrosis factor-α expression suggests modulation of central pro-inflammatory signaling pathways by rabeprazole.
- Rabeprazole improved systemic oxidative stress parameters by reducing lipid peroxidation and enhancing antioxidant enzyme activity.
- Combined administration of rabeprazole and prednisolone did not provide additive benefit, suggesting complex pharmacodynamic interactions.

## Introduction

Ulcerative colitis (UC) is a chronic inflammatory bowel disease characterized by relapsing inflammation of the colonic mucosa, leading to progressive tissue injury and impaired intestinal barrier function. Despite advances in therapeutic strategies, a substantial proportion of patients experience inadequate disease control, adverse effects, or loss of response to conventional treatments. This highlights the need to further elucidate mechanisms contributing to mucosal injury and to identify adjunctive therapeutic approaches.^1^^,^^2^

Oxidative stress plays a pivotal role in the pathogenesis of UC through excessive production of reactive oxygen species and insufficient antioxidant defense.^3^^,^^4^ Accumulating evidence indicates that oxidative stress not only amplifies inflammatory signaling but also promotes epithelial damage and persistence of mucosal injury.^3^^,^^5^ In particular, oxidative DNA damage, commonly reflected by increased levels of 8-hydroxy-2′-deoxyguanosine (8-OHdG), has been implicated in chronic inflammation and tissue remodeling in UC.^3^^,^^6^ These processes are closely linked to activation of key inflammatory pathways, including nuclear factor-κB (NF-κB), which regulates the transcription of proinflammatory cytokines such as tumor necrosis factor-α (TNF-α).^7^^,^^8^

Proton pump inhibitors (PPIs) are widely used for acid-related gastrointestinal disorders; however, emerging evidence indicates that certain PPIs may exert anti-inflammatory and antioxidant effects independent of gastric acid suppression.^9^^,^^10^ Experimental studies have reported that PPIs can modulate oxidative stress parameters, inhibit inflammatory cytokine production, and influence NF-κB signaling. Nevertheless, data regarding their potential role in inflammatory bowel disease remain limited and controversial, with most studies focusing on indirect clinical associations rather than direct experimental evidence.^11^^,^^12^

Rabeprazole, a PPI with unique pharmacokinetic and chemical properties, has been shown to exhibit antioxidant activity and immunomodulatory effects in various experimental settings.^9^^,^^10^ However, its impact on colonic inflammation, oxidative stress, and DNA damage in UC has not been fully elucidated.^13^ Moreover, comparative data evaluating its effects relative to established anti-inflammatory therapies, such as corticosteroids,^14^^,^^15^ are scarce.

Therefore, the current study aimed to investigate the effects of rabeprazole on histopathological injury, oxidative stress parameters, and inflammatory signaling pathways in an acetic acid–induced experimental model of UC. In addition, the efficacy of rabeprazole was compared with that of prednisolone, and the interaction of rabeprazole with prednisolone was evaluated.

## Materials and Methods

### Animals and Experimental Design

A total of 40 male Wistar albino rats (200-250 g) were obtained from Sivas Cumhuriyet University Animal Application and Research Center (DUAM). Animals were housed in a sound-insulated room at 22 ± 2°C with 55 ± 5% humidity and a 12-hour light/dark cycle. They were provided with standard pellet food and water ad libitum and were acclimatized for 7 days before the start of the experiment. Animals were monitored daily in accordance with these institutional animal care guidelines, and no animals were excluded from the final analysis.

Rats were randomly assigned into 5 groups (n = 8 per group) using a computer-generated random number sequence: control, acetic acid–induced colonic injury (AAIC), AAIC + rabeprazole, AAIC + prednisolone, and AAIC + rabeprazole + prednisolone. The experimental period lasted 10 days, after which the animals were anesthetized with ketamine and killed by cervical dislocation under deep anesthesia for biochemical and histopathological analyses. The 10-day treatment period was selected in accordance with established models evaluating the progression of colonic injury and early tissue recovery.^16^^,^^17^ This duration is consistent with the literature on short-term pharmacological interventions in experimental colitis^18^ and provides a sufficient window to assess the pleiotropic anti-inflammatory and tissue-repair effects of rabeprazole.^9^^,^^10^

Macroscopic, histopathological, and immunohistochemical evaluations were performed by an experienced pathologist blinded to the experimental group assignments. To ensure objectivity, all slides were coded, and the pathologist remained unaware of the group allocations until the evaluation of the entire study was completed. A similar approach was used for the biochemical analyses as well.

### Induction of Experimental Acetic Acid–Induced Colonic Injury

AAIC was induced by transrectal administration of 2 mL of 3% acetic acid using a flexible polyurethane catheter inserted approximately 4.5 cm into the rectum.^19^^,^^20^ To ensure uniform distribution within the distal colon and prevent leakage, rats were maintained in the Trendelenburg position for 1 minute after instillation.

### Drug Administration

Rabeprazole (20 mg/kg/day) and prednisolone (2 mg/kg/day) were administered orally by gavage once daily for 10 consecutive days, starting on the day of colitis induction. For combination administration, both drugs were administered at the same doses and schedule. The dose of rabeprazole (20 mg/kg) was justified based on prior experimental studies demonstrating its pleiotropic anti-inflammatory effects in rodents^9^^,^^10^ and corresponds to an allometrically scaled dose used to evaluate non-gastric targets.^10^ Rabeprazole (Bilim İlaç, Kocaeli, Türkiye) and prednisolone (, Deva, İstanbul, Türkiye) were prepared daily by dissolving the drugs in 0.9% NaCl. To eliminate potential confounding factors related to handling stress, the control and AAIC groups received a sham gavage of 1 mL of 0.9% NaCl on the same schedule as the treatment groups. The primary endpoint was defined as the total histopathological damage score.

### Assessment of Oxidative Stress Parameters

Serum levels of malondialdehyde (MDA), glutathione (GSH), superoxide dismutase (SOD), and catalase (CAT) were measured using commercially available enzyme-linked immunosorbent assays kits according to the manufacturers’ instructions.

### Macroscopic and Histopathological Evaluation

Following euthanasia, colonic tissues were excised and examined macroscopically. Macroscopic damage was scored using the Wallace scoring system.^21^

For histopathological evaluation, colon samples were fixed in 10% neutral-buffered formalin, embedded in paraffin, and sectioned at 5 μm thickness. Sections were stained with hematoxylin and eosin. Histological damage, including ulceration, mononuclear cell infiltration, crypt atrophy, subepithelial hemorrhage, and edema, was assessed semiquantitatively on a scale of 0 (absent) to 3 (severe), as previously described.

### Immunohistochemical Analysis

Paraffin-embedded colon sections were processed for immunohistochemical staining using standard protocols. Five-micrometer sections mounted on poly-L-lysine-coated slides were deparaffinized and rehydrated through xylene and graded alcohol series. Endogenous peroxidase activity was blocked with 3% H_2_O_2_ for 10 minutes, followed by heat-induced antigen retrieval (2 × 5 minutes at 500 W). Sections were then incubated overnight with primary antibodies against 8-OHdG (Santa Cruz, sc-66036, Biotechnology, Dallas, Texas, USA), transforming growth factor-β (TGF-β; Santa Cruz, sc-130348, Biotechnology, Dallas, Texas, USA), TNF-α (Santa Cruz, sc-133192, Biotechnology, Dallas, Texas, USA), and NF-κB (Abcam, ab7971, Cambridge, UK) at optimal dilutions. Immunodetection was performed using a HRP-conjugated antipolyvalent detection system (Thermo Fisher Scientific, TP-125-HL, Waltham, USA) and visualized with diaminobenzidine chromogen. After counterstaining with Mayer’s hematoxylin and mounting, immunoreactivity was evaluated semiquantitatively on a scale of 0 (absent) to 4 (very severe).

### Statistical Analysis

Statistical analyses were performed using Statistical Package for the Social Sciences 22.0 (IBM SPSS Corp.; Armonk, NY, USA). Normality was assessed using the Shapiro–Wilk test (n = 8). Parametric serum parameters (MDA, GSH, SOD, and CAT) were analyzed using 1-way analysis of variance followed by Tukey post hoc test (mean ± SD). Nonparametric histopathological and immunohistochemical scores were analyzed using Kruskal–Wallis and post-hoc Mann–Whitney *U*-tests with Bonferroni correction (median [interquartile range (IQR)]). A *P* value of less than .05 was considered statistically significant. Effect size was determined using eta-squared (*η*^2^), and significant intergroup differences were indicated in tables using a superscript lettering system.

### Ethics Approval

All experimental procedures were conducted in accordance with the *Guide for the Care and Use of Laboratory Animals* and were approved by the Animal Experiments Local Ethics Committee of Sivas Cumhuriyet University (Approval No: 65202830-050.04.04-772, Date: October 20, 2023). This study is retrospective and therefore did not require informed consent. This study used Open AI to improve language and edit grammar during the preparation of the manuscript.

## Results

### Macroscopic and Histopathological Findings

**Figure 1.**
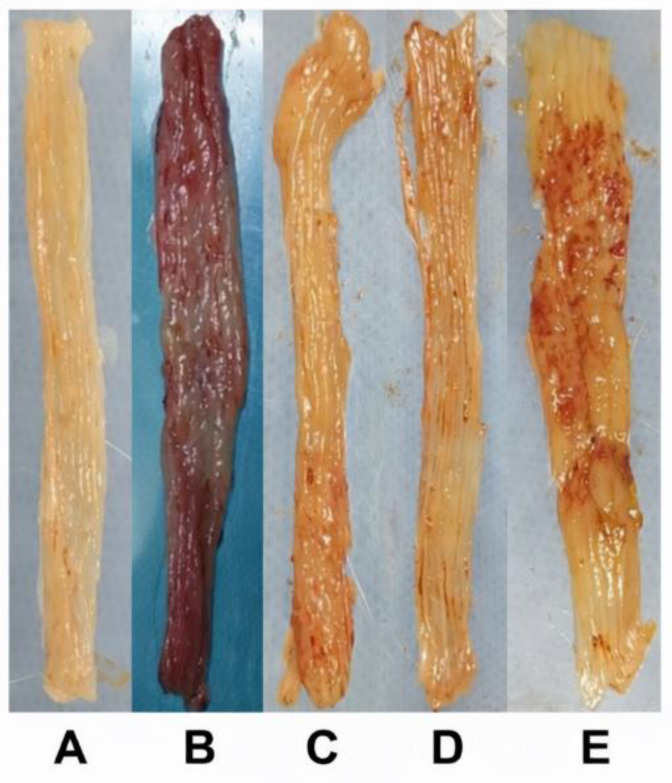
Representative macroscopic appearances of colonic tissue from control (A), acetic acid–induced colonic injury (AAIC; B), AAIC + rabeprazole (C), AAIC + prednisolone (D), and AAIC + rabeprazole + prednisolone (E) groups. Severe ulcerative lesions are evident in the AAIC group, whereas rabeprazole- and prednisolone-treated groups exhibit mild hyperemic changes. The combined treatment group demonstrates moderate ulcerative lesions.

Macroscopic evaluation of colonic tissues showed a normal appearance in the control group, whereas extensive ulceration and mucosal damage were observed in the AAIC group. Rats treated with rabeprazole or prednisolone demonstrated markedly reduced macroscopic injury compared with the AAIC group. In contrast, combined administration of rabeprazole and prednisolone did not result in further macroscopic improvement and showed intermediate injury severity (Figure 1).

**Figure 2.**
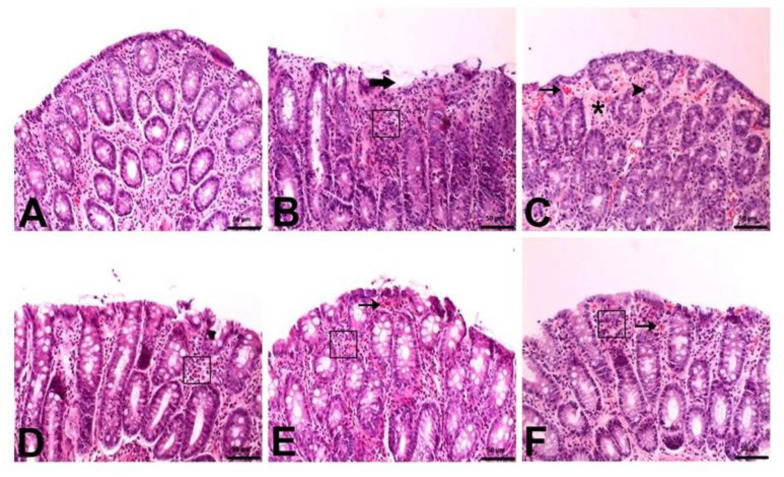
Representative histopathological features of colonic tissue sections stained with hematoxylin and eosin. Panel A shows normal histological architecture in the control group. Panels B and C (acetic acid–induced colonic injury [AAIC] group) demonstrate severe ulceration (arrow), marked mononuclear cell infiltration (boxed area), moderate crypt atrophy (arrowhead), subepithelial hemorrhage (thin arrow), and subepithelial edema (asterisk). Panel D (AAIC + rabeprazole) shows mild mononuclear cell infiltration. Panel E (AAIC + prednisolone) demonstrates mild mononuclear cell infiltration with focal subepithelial hemorrhage. Panel F (AAIC + rabeprazole + prednisolone) shows moderate mononuclear cell infiltration with mild subepithelial hemorrhage.

Histopathological examination revealed significant differences among groups in crypt damage, ulceration, mononuclear cell infiltration, hemorrhage, and edema (*P* < .001). The AAIC group demonstrated severe crypt damage, mucosal ulceration, and pronounced mononuclear inflammatory cell infiltration (median [IQR]: 3.00 [3.00-3.00]for all parameters). Administration of rabeprazole or prednisolone significantly reduced inflammatory cell infiltration, crypt damage, edema, and ulceration scores compared with the AAIC group (*P *= .0002). Notably, rabeprazole monotherapy was significantly more effective than prednisolone in preventing hemorrhage, with scores comparable to those of the control group (*P *= .0002 vs. combination group). In the combination group, inflammatory cell infiltration remained significantly lower than in the AAIC group (*P *= .0002) but was significantly higher than in either the rabeprazole (*P *= .0006) or prednisolone (*P *= .0001) monotherapy groups (Table 1, Figure 2).

### Immunohistochemical Analysis

Immunohistochemical evaluation of oxidative and inflammatory markers demonstrated marked differences among groups (*P* < .001 for all comparisons). Minimal immunoreactivity for 8-OHdG, TGF-β, TNF-α, and NF-κB was observed in the control group (median [IQR]: 1.00 [1.00-1.25]). In contrast, the AAIC group exhibited intense immunopositivity for all markers (median [IQR]: 4.00 [3.75-4.00]; *P* < .001).

**Figure 3.**
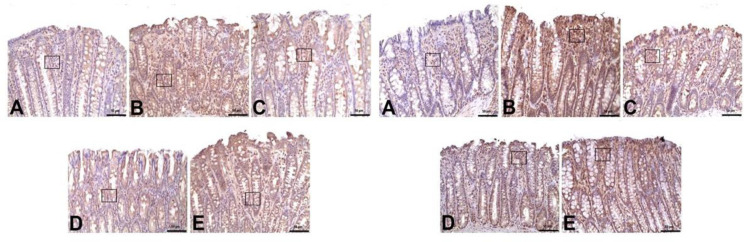
Representative immunohistochemical staining of nuclear factor-κB (left panels) and tumor necrosis factor-α (right panels) in colonic tissue sections from control (A), acetic acid–induced colonic injury (AAIC; B), AAIC + rabeprazole (C), AAIC + prednisolone (D), and AAIC + rabeprazole + prednisolone (E) groups. Mild immunoreactivity is observed in the control group, whereas intense nuclear and cytoplasmic staining is evident in the AAIC group. Rabeprazole- and prednisolone-treated groups show moderate immunoreactivity, whereas combined treatment is associated with severe staining. Boxed areas indicate representative fields used for immunohistochemical evaluation. Original magnification ×200.

**Figure 4.**
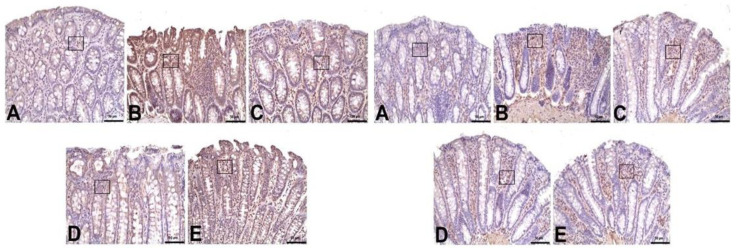
Representative immunohistochemical staining of 8-hydroxy-2′-deoxyguanosine (left panels) and transforming growth factor-β (right panels) in colonic tissue sections from control (A), acetic acid–induced colonic injury (AAIC; B), AAIC + rabeprazole (C), AAIC + prednisolone (D), and AAIC + rabeprazole + prednisolone (E) groups. Mild immunoreactivity is observed in the control group, whereas intense nuclear and cytoplasmic staining is evident in the AAIC group. Rabeprazole- and prednisolone-treated groups show moderate immunoreactivity, whereas combined treatment is associated with severe staining. Boxed areas indicate representative fields used for immunohistochemical evaluation. Original magnification ×200.

Both rabeprazole and prednisolone significantly reduced the immunohistochemical expression of 8-OHdG, TGF-β, TNF-α, and NF-κB (median [IQR]: 2.00 [2.00-2.25]) compared with the AAIC group (*P* = .0006). The degree of reduction was similar in the rabeprazole and prednisolone groups. In the combination group, immunopositivity for all markers remained significantly lower than in the AAIC group (median [IQR]: 3.00 [3.00-3.00]; *P* = .0002) but was higher than in either the rabeprazole (*P* = .0033) or prednisolone (*P* = .0008) monotherapy groups (Table 2, Figures 3 and 4), indicating a lack of additive effect with combined administration.

### Oxidative Stress Parameters

**Figure 5.**
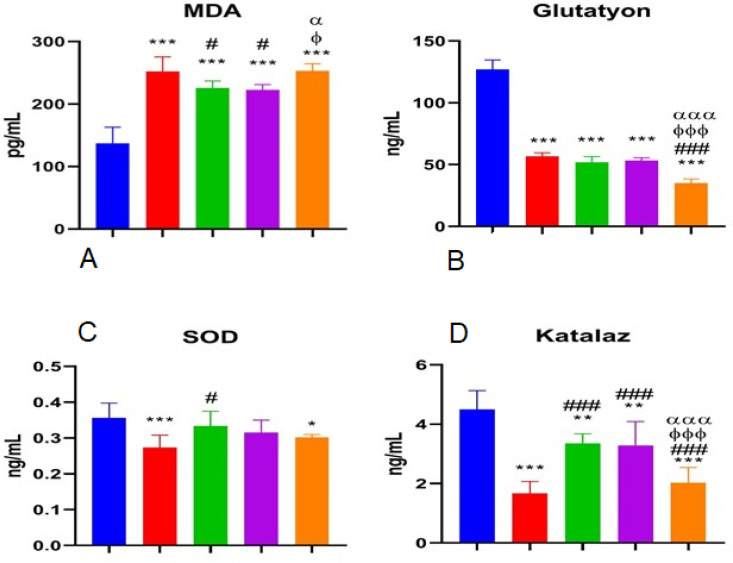
Serum levels of (A) malondialdehyde, (B) glutathione, (C) superoxide dismutase, and (D) catalase in the control (blue), acetic acid–induced colonic injury (AAIC) (red), AAIC + rabeprazole (green), AAIC + prednisolone (purple), and AAIC + rabeprazole + prednisolone (orange) groups. Data are presented as mean ± SD. Statistical significance was determined using 1-way analysis of variance followed by Tukey’s post hoc test. * *P* < .05, ** *P* < .01, *** *P* < .001 vs. the control group; # *P* < .05, ### *P* < .001 vs. the AAIC group; φ *P* < .05, φφφ *P* < .001 for AAIC + rabeprazole vs. AAIC + rabeprazole + prednisolone; α *P* < .05, ααα *P* < .001 for AAIC + prednisolone vs. AAIC + rabeprazole + prednisolone.

Serum oxidative stress analysis revealed significant differences across experimental groups. MDA levels were significantly increased in the AAIC group compared with the control group (*P* < .001). Administration of rabeprazole and prednisolone significantly reduced MDA levels relative to the AAIC group (*P* < .05). In the combination group, MDA levels were significantly higher than those observed in the rabeprazole and prednisolone groups (Figure 5).

SOD and CAT activities were significantly decreased in the AAIC group compared with the control group (*P* < .001). Both rabeprazole and prednisolone partially restored SOD and CAT activities compared with the AAIC group (*P *< .05 and *P* < .01, respectively). In the combination group, antioxidant enzyme activities were higher than in the AAIC group but lower than in the rabeprazole and prednisolone groups (*P *< .05 and *P* < .001, respectively; Figure 5).

GSH levels were significantly reduced in all colonic injury–induced groups compared with the control group (*P* < .001). The lowest GSH levels were observed in the combination group and were significantly lower than those in the rabeprazole and prednisolone groups (*P* < .001; Figure 5).

## Discussion

The current study examined whether rabeprazole modulates oxidative stress–related and inflammatory pathways in an acetic acid–induced experimental model of colonic injury and compared these effects with those of prednisolone. The results indicate that rabeprazole administration was associated with attenuation of colonic tissue injury, modulation of oxidative stress parameters, and downregulation of selected inflammatory and DNA damage markers.

Oxidative stress is recognized as a central contributor to mucosal injury in AAIC through excessive generation of reactive oxygen species and impairment of endogenous antioxidant defenses.^3^^,^^22^^,^^23^ Consistent with previous experimental studies, colitis induction in the current model resulted in elevated lipid peroxidation, reflected by increased MDA levels, together with reduced antioxidant enzyme activities and GSH depletion. Administration of rabeprazole was associated with partial normalization of selected oxidative stress parameters.

The reduction in 8-OHdG immunoreactivity observed in rabeprazole-treated animals indicates modulation of oxidative DNA damage, a recognized downstream consequence of sustained oxidative stress in inflammatory bowel disease. Similar reductions in 8-OHdG levels have been reported in experimental colitis models following pharmacological modulation of redox-sensitive pathways.^24^^,^^25^ Given the association between DNA damage, persistent mucosal injury, and long-term complications, this finding is mechanistically relevant in the context of AAIC.^23^ Consistent with the recent literature, the suppression of oxidative stress and inflammatory signaling pathways plays a critical role in restoring the intestinal mucosal barrier and resolving tissue damage.^26^^,^^27^

Recent large-scale population-based cohort studies and meta-analyses have suggested that regular PPI therapy may be associated with adverse outcomes and an increased risk of IBD development. For instance, Deleuran et al^28^ and Fossmark et al^29^ reported that long-term acid suppression could negatively influence the disease course in clinical settings. A meta-analysis by Zhang et al^30^ further identified an association between PPI exposure and increased risk of adverse events in IBD patients. These detrimental clinical effects have been primarily attributed to gut microbiota dysbiosis and increased susceptibility to enteric pathogens. Consistent with this, Cao et al^31^ reported that PPIs can alter the microbial landscape implicated in the pathogenesis of UC.

Although these large-scale cohorts^28^^,^^29^ and meta-analyses^30^ evaluate PPIs as a single class and associate long-term acid suppression with IBD risk via gut dysbiosis, such class-wide assessments may overlook molecule-specific immunomodulatory properties.^9^^,^^10^ Although PPIs have been reported to induce dysbiotic shifts,^31^ their ability to modulate the immune system through the inhibition of specific proinflammatory cytokines and transcriptional cascades (e.g., NF-κB) may provide a distinct therapeutic advantage in inflammatory conditions. Indeed, the results demonstrated that inflammatory signaling pathways were also affected by rabeprazole. Decreased immunohistochemical expression of TNF-α and NF-κB indicates suppression of central proinflammatory transcriptional cascades involved in intestinal inflammation.^7^^,^^32^ The magnitude of these changes showed partial overlap with those observed following prednisolone administration, suggesting that rabeprazole may modulate selected components of inflammatory signaling rather than acting as a conventional anti-inflammatory agent. The net effect of certain PPIs such as rabeprazole on colonic injury appears to reflect a balance between their potential for dysbiosis and their intrinsic anti-inflammatory activities. In the current AAIC model, the direct antioxidant and anti-inflammatory properties of rabeprazole, mediated through the suppression of NF-κB and TNF-α, appear to reflect the state before long-term microbiota-induced risks emerge. These findings are consistent with those of Gandhi et al^18^ who demonstrated that lansoprazole prevents IBD in rats by reducing oxidative stress and nitric oxide levels, supporting the hypothesis that PPIs may exert protective effects during the acute phase of mucosal injury. Therefore, although long-term acid suppression has well-documented clinical risks, the intrinsic molecular mechanisms of rabeprazole may offer mucosal protection against acute oxidative injury.

Notably, the combined administration of rabeprazole and prednisolone did not produce additive effects. Across histopathological, oxidative stress, and immunohistochemical parameters, the protective responses observed with monotherapy were reduced when both agents were administered together. Although the current study was not designed to determine the mechanistic basis of this interaction and the underlying molecular mechanisms remain unclear, these findings warrant further investigation, given the common clinical practice of co-prescribing PPIs and corticosteroids. This lack of additivity may reflect complex pharmacodynamic interactions or shared signaling feedback loops that limit cumulative benefit. The concurrent use of these agents should therefore be approached with caution in clinical practice until underlying mechanistic interactions are further clarified through molecular studies.

Taken together, these results demonstrate that rabeprazole modulates selected oxidative stress–related and inflammatory pathways in AAIC. However, its effects do not appear to be additive when combined with prednisolone, highlighting the complexity of drug interactions within inflammatory signaling networks.^33^

The current study has several limitations. An acute experimental colitis model was used, and neither dose–response relationships nor long-term outcomes were evaluated.^17^^,^^34^ Furthermore, the lack of longitudinal clinical measures, such as daily body weight change and stool consistency scores, limits the assessment of clinical severity correlation. Further studies incorporating chronic models and detailed molecular pathway analyses are warranted to clarify the mechanistic profile of rabeprazole in intestinal inflammation.

In conclusion, rabeprazole exerts significant mucosal protection against AAIC by modulating oxidative stress and suppressing NF-κB-mediated inflammatory signaling, indicating pleiotropic effects beyond acid suppression. However, these beneficial effects were not additive when combined with prednisolone, suggesting a potential pharmacodynamic interaction. These findings highlight the complexity of drug–drug interactions within inflammatory pathways and warrant a cautious approach to the clinical co-administration of PPIs and corticosteroids until the underlying mechanisms are fully elucidated. Furthermore, these results provide mechanistic insight with potential translational relevance for future studies of adjunct therapeutic strategies in intestinal inflammation.

## Figures and Tables

**Table 1.** Semiquantitative Histopathological Scores of Colonic Tissue in the Experimental Groups

**Group**	**Ulceration**	**Mononuclear Cell Infiltration**	**Crypt Atrophy**	**Hemorrhage**	**Edema**
Control	0.00 (0.00-0.00)^a^	0.00 (0.00-0.00)^a^	0.00 (0.00-0.00)^a^	0.00 (0.00-0.00)^a^	0.00 (0.00-0.00)^a^
AAIC	3.00 (3.00-3.00)^b^	3.00 (3.00-3.00)^b^	3.00 (2.75-3.00)^b^	2.00 (2.00-2.00)^c^	2.00 (2.00-2.00)^b^
AAIC + Rab	0.00 (0.00-0.00)^a^	1.00 (1.00-1.25)^c^	0.00 (0.00-0.00)^a^	0.00 (0.00-0.00)^a^	0.00 (0.00-0.00)^a^
AAIC + Pred	0.00 (0.00-0.00)^a^	1.00 (1.00-1.00)^c^	0.00 (0.00-0.00)^a^	1.00 (1.00-1.25)^b^	0.00 (0.00-0.00)^a^
AAIC + Rab + Pred	0.00 (0.00-0.00)^a^	2.00 (2.00-2.00)^d^	0.00 (0.00-0.00)^a^	1.00 (1.00-1.00)^b^	0.00 (0.00-0.00)^a^
P*	<.001	<.001	<.001	<.001	<.001

For all histopathological parameters analyzed, the Kruskal–Wallis test statistic (*χ*^2^) ranged from 34.41 to 38.79, and the effect size (*η*^2^) was found to be between 0.86 and 0.99. These values confirm that the statistical power and experimental effect of the differences between groups are very high (*P* < .001).

AAIC, acetic acid–induced colonic injury; Pred, prednisolone; Rab, rabeprazole.

^*^The Kruskal–Wallis test was used for the overall intergroup comparison. Data are presented as the median (interquartile range).

a, b, c, d: Differences between groups carrying different superscript letters in the same column are statistically significant according to the Mann–Whitney *U*-test (with Bonferroni correction) (*P* < .05).

**Table 2.** Semiquantitative Immunohistochemical Scores of Oxidative and Inflammatory Markers

**Group**	**8-OHdG**	**TGF-β**	**TNF-α**	**NF-κB**
Control	1.00 (1.00-1.00)ᵃ	1.00 (1.00-1.00)ᵃ	1.00 (1.00-1.00)ᵃ	1.00 (1.00-1.25)ᵃ
AAIC	4.00 (4.00-4.00)ᵇ	4.00 (3.75-4.00)ᵇ	4.00 (4.00-4.00)ᵇ	4.00 (4.00-4.00)ᵇ
AAIC + Rab	2.00 (2.00-2.25)ᶜ	2.00 (2.00-2.25)ᶜ	2.00 (2.00-2.00)ᶜ	2.00 (2.00-2.25)ᶜ
AAIC + Pred	2.00 (2.00-2.00)ᶜ	2.00 (2.00-2.00)ᶜ	2.00 (2.00-3.00)ᶜ	2.00 (2.00-2.25)ᶜ
AAIC + Rab + Pred	3.00 (3.00-3.00)ᵈ	3.00 (3.00-3.00)ᵈ	3.00 (3.00-3.00)ᵈ	3.00 (3.00-3.00)ᵈ
P*	<.001	<.001	<.001	<.001

For all immunohistochemical parameters analyzed, the Kruskal–Wallis test statistic (*χ*^2^) ranged from 32.38 to 35.44, and the effect size (*η*^2^) ranged from 0.81 to 0.90. These values confirm that the observed immunohistochemical changes and administration responses have a statistically very strong effect (*P* < .001).

8-OHdG, 8-hydroxy-2′-deoxyguanosine; AAIC, acetic acid–induced colonic injury; NF-κB, nuclear factor-kappa B; Pred, prednisolone; TGF-β, transforming growth factor-beta; TNF-α, tumor necrosis factor-alpha; Rab, rabeprazole.

^*^The Kruskal–Wallis test was used for the overall intergroup comparison. Data are presented as the median (interquartile range).

a, b, c, d: Differences between groups carrying different superscript letters in the same column are statistically significant according to the Mann–Whitney *U*-test (with Bonferroni correction) (*P* < .05).

## Data Availability

The data that support the findings of this study are available on request from the corresponding author.
